# Bidirectional transfer of a small membrane-impermeable molecule between the *Caenorhabditis elegans* intestine and germline

**DOI:** 10.1016/j.jbc.2024.107963

**Published:** 2024-11-05

**Authors:** Sarah Turmel-Couture, Pier-Olivier Martel, Lucie Beaulieu, Xavier Lechasseur, Lloyd Venceslas Fotso Dzuna, Patrick Narbonne

**Affiliations:** Département de Biologie Médicale, Université du Québec à Trois-Rivières, Trois-Rivières, Quebec, Canada

**Keywords:** *Caenorhabditis elegans*, germline, intestine, sheath cells, stem cells, gap junctions, endocytosis, cell signaling, ERK/MAPK, 5-carboxyfluorescein

## Abstract

The extracellular signal-regulated kinase/mitogen-activated protein kinase (ERK/MAPK) is a positive regulator of cell proliferation often upregulated in cancer. Its *Caenorhabditis elegans* ortholog MPK-1 stimulates germline stem cell (GSC) proliferation nonautonomously from the intestine or somatic gonad. How MPK-1 can perform this task from either of these two tissues however remains unclear. We reasoned that somatic MPK-1 activity could lead to the generation of proproliferative small molecules that could transfer from the intestine and/or somatic gonad to the germline. Here, in support of this hypothesis, we demonstrate that a significant fraction of the small membrane-impermeable fluorescent molecule, 5-carboxyfluorescein, transfers to the germline after its microinjection in the animal’s intestine. The larger part of this transfer targets oocytes and requires the germline receptor mediated endocytosis 2 (RME-2) yolk receptor. A minor quantity of the dye is however distributed independently from RME-2 and more widely in the animal, including the distal germline, gonadal sheath, coelomocytes, and hypodermis. We further show that the intestine-to-germline transfer efficiency of this RME-2 independent fraction does not vary together with GSC proliferation rates or MPK-1 activity. Therefore, if germline proliferation was influenced by small membrane-impermeable molecules generated in the intestine, it is unlikely that proliferation would be regulated at the level of molecule transfer rate. Finally, we show that conversely, a similar fraction of germline injected 5-carboxyfluorescein transfers to the intestine, demonstrating transfer bidirectionality. Altogether, our results establish the possibility of an intestine-to-germline signaling axis mediated by small membrane-impermeable molecules that could promote GSC proliferation cell nonautonomously downstream of MPK-1 activity.

The transfer of information between adjacent and distant cells is important for many biological processes in multicellular organisms, including survival, proliferation, differentiation, and cell migration (1, 2). Accordingly, defects in information transfer contribute to several diseases, including cancer (3, 4, 5). Among the various types of intertissular interactions, soma-germline communications are critical to support gamete development and coordinate progeny production (6, 7, 8, 9).

*Caenorhabditis elegans* is an excellent simple model to study soma-germline interactions. In hermaphrodites, the germline is comprised within two anterior and posterior symmetrical U-shaped somatic gonadal arms that share a common uterus. Distinct germline stem cell (GSC) populations are found at the distal end of each gonad arm where GSCs have two possible fates: they can proliferate to expand in numbers or differentiate to become gametes. This decision is controlled by the expression of a Notch ligand, LAG-2, by two distal tip cells, one per distal gonad arm extremity (7, 10, 11). GSCs will therefore proliferate until their daughter cells are displaced beyond this niche signal range and launch their differentiation program (12). The distal germline region encompassing the GSCs and their proliferating progeny is termed the progenitor zone (12, 13, 14). Germ cells therefore mature, progressing through meiosis as they move along the distal-proximal axis of the gonad to eventually turn into sperm during larval stages and into oocytes during adulthood. As part of the somatic gonad, five pairs of sheath cells assemble in a thin layer around each arm and are critical for germline growth, organization, and function (7, 9, 15). Interestingly, the innexins that localize to the soma-germline interface are required for GSC proliferation and differentiation, including for oocyte maturation (16, 17, 18, 19) and as such, they may mediate a large part of the essential soma-germline interactions. In addition to requiring gap junction connections with the somatic gonad, GSC proliferation is stimulated by two parallel pathways (20, 21, 22, 23). On the one hand, nutrition promotes GSC proliferation by activating the insulin/IGF-1 signaling (IIS) pathway within GSCs (20, 21, 24), and on the other, the extracellular signal-regulated kinase (ERK)/mitogen-activated protein kinase MPK-1 nonautonomously promotes GSC proliferation from the animal’s intestine or somatic gonad (23, 25).

While the way by which IIS promotes GSC proliferation cell autonomously may conceptually appear straightforward, how MPK-1 promotes GSC proliferation at a distance, from either of two tissues, is much less clear. We reasoned that small signaling molecules and/or building blocks must transfer from the animal’s intestine or somatic gonad toward the germline downstream of MPK-1 activity to nonautonomously stimulate germline proliferation. Since GSC proliferation can be supported by MPK-1 activity in either the intestine or the somatic gonad, the proliferation stimulating molecules could be shared between both somatic tissues, and hence be produced or released by either of them, from where they must reach the germline, or minimally make contact with surface receptors.

If the critical small molecules are membrane-impermeable, this hypothesis would depend on the presence of a functional coupling between the *C. elegans* intestine and germline. Intestinal cells are coupled together by INX-16 junctions (26), while the somatic gonad expresses INX-8/INX-9 hybrid hemichannels that connect it to the germline *via* INX-14/INX-21 and INX-14/INX-22 hybrid hemichannels in the distal and proximal germline, respectively (13, 18). Although both the intestine and the somatic gonad express innexins, there is no evidence for a direct coupling of the intestine to the somatic gonad *via* gap junctions since the two tissues do not adhere to each other and are separated by pseudocoelomic fluids (9, 19, 27). Nonetheless, membrane-impermeable molecules can be transferred across the pseudocoelom *via* exo/endocytosis. For example, yolk is synthesized in the intestine and secreted in the pseudocoelomic space to be actively uptaken by the growing oocytes *via* receptor-mediated endocytosis (28). As such, we evaluated here the diffusion pattern of a small membrane-impermeable fluorescent molecule, 5-carboxyfluorescein (5-CF), following its microinjection into the animal's intestine and germline. We found that a significant fraction of 5-CF transferred from the intestine to the germline and *vice versa*. Moreover, other tissues of the animal showed relatively low fluorescence, suggesting the existence of a bidirectional small membrane-impermeable molecule exchange between the intestine and germline that could potentially be implicated in the regulation of GSC proliferation.

## Results

### A small membrane-impermeable molecule transfers from the intestine to the germline

Innexin-based gap junctions allow for the passage of small molecules and ions of up to 2 kDa (18, 29). We selected 5-CF, a small membrane-impermeable (30) green fluorescent dye having a molecular size of 0,376 kDa, as a molecule that could diffuse form one cell to another through gap junctions but not through the cell membrane. Moreover, 5-CF was successfully used to visualize diffusion through pharyngeal innexins in *C. elegans* (31).

**Figure 1:**
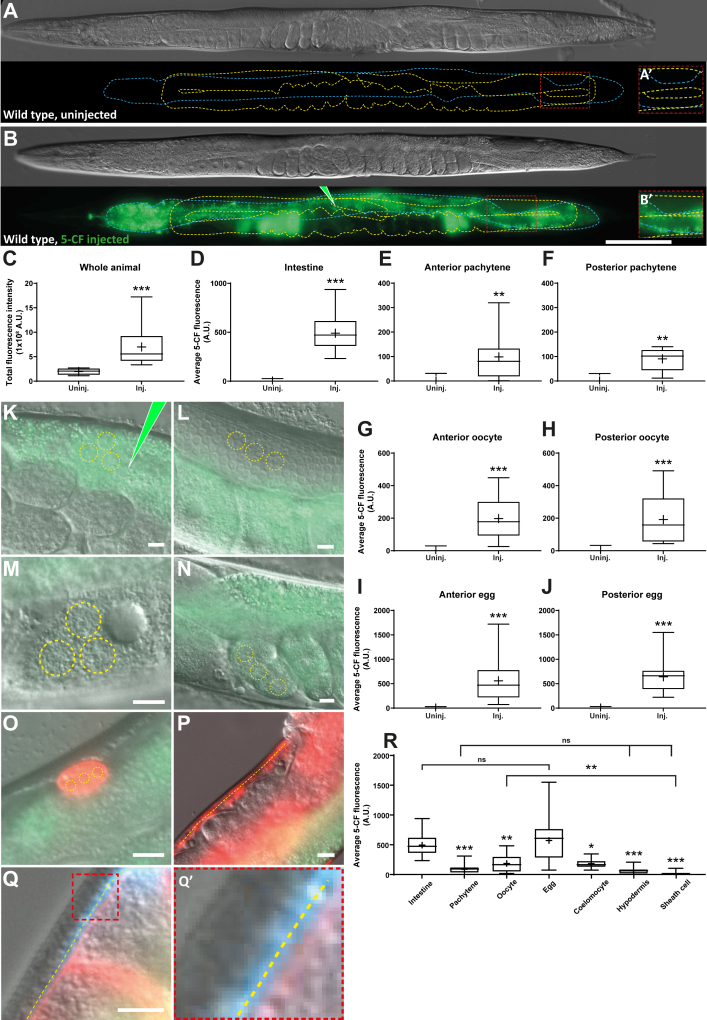
**A small membrane-impermeable molecule transfers from the intestine to the germline.***A* and *B*, representative DIC images and fluorescence maximal projections of WT adults that were or were not microinjected with 5-CF in the intestine, near the vulva (*green* pipette tip). The intestine is delimited by the *blue dashed line* while the germline is delimited by a *yellow dashed line*. All display images were thresholded to remove background autofluorescence (see experimental procedures). The scale bar represents 100μm. Posterior germline pachytene regions were enlarged and enhanced in A’-B’ to allow visualization of the dimmer signal present in this area, where applicable. *C*, total fluorescence intensity measured in the whole animal (see Experimental procedures). The signal present in uninjected controls represents the background autofluorescence. *D*, average intestinal fluorescence intensity measured near the injection site. From here onward, background autofluorescence was subtracted from both injected and uninjected controls. *E*–*J*, average 5-CF fluorescence intensities measured in the different anterior and posterior germline regions indicated. *K*–*Q*, DIC-fluorescence overlay images of all analyzed regions are shown to illustrate how quantifications were performed. All displayed animals are WT adults that were injected in the intestine, near the vulva. For the (*K*) intestine, (*L*) pachytene region, (*M*) −1 oocyte, (*N*) +1 egg, and (*O*) coelomocytes (marked by *coel::RFP*), the mean intensity was measured by averaging up to three circular zones (*yellow dashed circles*) per region when it was in focus (See Experimental procedures). For the (*P*) hypodermis (marked by *mKate2*) and (*Q*) proximal sheath cells (marked by *TagBFP2*; germ membranes are marked with *TagRFP-T*; enlarged in *Q’*), up to two longitudinal zones (*yellow dashed lines*) were averaged per animal, one in the anterior half and one in the posterior half. The scale bars represent 10 μm. *R*, intertissue comparison of the average 5-CF fluorescence intensity measured in all the different regions analyzed. Anterior and posterior values were averaged where applicable. *C*–*J*, and *R*, sample sizes, *C* and *D*: 9, 23; *E*: 7, 12; *F*: 6, 7; *G*: 9, 12; *H*: 8, 14; *I*: 8, 21; *J*: 7, 20; *R*: 23, 19, 26, 40, 27, 34, 11. Statistical tests, *C*: Mann–Whitney; *D*, *E*, *G*–*J*: Welch’s; *F*: *t* test; *R*: Kruskal–Wallis. 5-CF, 5-carboxyfluorescein; DIC, differential interference contrast.

We first asked whether 5-CF could transfer from the intestine to the germline. We microinjected 30 to 50 pL (32) of a saturated 5-CF solution (31) in the intestine of WT adult hermaphrodites near the vulva, and allowed animals to recover for an hour before imaging them (Fig. 1, *A* and *B*) (see Experimental procedures for details). We then measured the relative fluorescence intensity in the whole animal, in the intestine near the injection site, as well as in the following parts of the germline in each gonad arm: pachytene region, proximal oocytes, and distal eggs (Fig. 1, *C*–*N*). By measuring whole animal fluorescence, we controlled that the injected 5-CF volume was sufficient to be easily detected after the recovery time and was well above the background autofluorescence (Fig. 1*C*). This also allowed to normalize the fluctuations in the injection volume between individuals. An intense fluorescence signal was detectable in the intestine after the recovery period, not only near the injection site but across the whole intestine (Fig. 1, *B* and *D*). A weaker signal was also detectable in the germline pachytene area (Fig. 1, *A*, *B*, *E* and *F*) and proximal oocytes (Fig. 1, *A*, *B*, *G* and *H*), and a strong signal was present in the distal eggs (Fig. 1, *A*, *B*, *I* and *J*). The presence of dye in the distal-most eggs, despite their impermeable eggshell, likely originates from uptake before they were fertilized as we expected the fertilization of 1 to 3 new oocytes per gonad arms to occur during the recovery period (15, 33). We draw two conclusions from this experiment. First, the roughly even spread of 5-CF throughout the intestine confirms that intestinal cells are interconnected by gap junctions that allow the dye to freely diffuse across the tissue (26, 27, 34). Second, since a significant fraction of 5-CF transferred from the intestine to the germline, we conclude that small membrane-impermeable molecules produced in the intestine can reach the germline and influence its activities.

The dye did not visibly accumulate into any other tissues of the animal as much as it did in the first few oocytes that became eggs over the recovery period, where its concentration reached that of the intestine (Fig. 1, *A*–*R*). However, to further evaluate the specificity of the intestine-to-germline transfer, we quantified 5-CF fluorescence in the hypodermis and coelomocytes, two highly endocytic tissues (35, 36), as well as in the gonadal sheath cells. We found that the coelomocytes, which nonspecifically endocytose pseudocoelomic fluids (36), accumulated 5-CF over the hour of recovery to levels that were similar to those attained within the proximal oocytes (Figs. 1, *O* and *R*, S1). The hypodermis and proximal sheath cells had lower 5-CF levels, comparable to within the germline pachytene region (Figs. 1, *P*–*R*, S1). We thus conclude that small membrane-impermeable molecules, such as 5-CF, can transfer rapidly, efficiently, and rather specifically from the intestine to the proximal germline (to the mature oocytes), while this may expose a specific signaling axis between these two tissues. Significant quantities of small membrane-impermeable molecules can also transfer less specifically from the intestine to other tissues of the animal, including the distal germline, gonadal sheath, coelomocytes, and hypodermis, where they can reach relatively smaller, yet significant concentrations.

### 5-CF does not transfer through intact membranes

**Figure 2:**
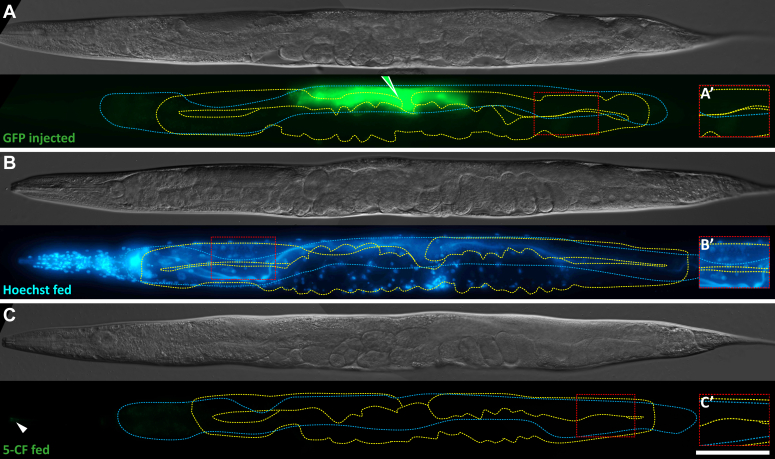
**A larger molecule does not transfer from the intestine to the germline.***A*–*C*, representative DIC images and fluorescence maximal projections of WT adults that were (*A*) microinjected with GFP in the intestine, near the vulva (green pipette tip), or soaked in (*B*) Hoechst or (*C*) 5-CF. The intestine is delimited by a *blue dashed line* while the germline is delimited by a *yellow dashed line*. The scale bar represents 100μm. Anterior or posterior germline pachytene regions were enlarged and enhanced in A’–C’ to allow visualization of the dimmer signal present in this area, where applicable. 5-CF, 5-carboxyfluorescein; DIC, differential interference contrast.

We designed several controls to ensure that 5-CF was indeed membrane-impermeable and that the diffusion pattern that we observed did not occur due to membrane disruptions during manipulations. First, we microinjected another membrane-impermeable fluorescent molecule that is too large to pass through gap junctions, the GFP, which has a relatively high molecular weight of 27 kDa. As expected, GFP microinjected in the intestine remained restricted within the few intestinal cells around the injection site where separating membranes likely burst by the injection blow, and it did not diffuse into farther intestinal cells, nor into any other tissues of the animal (Fig. 2*A*). This also shows that the hole left after the microinjection is immediately sealed as the microneedle is removed, and that the procedure does not lead to any significant leakage.

Next, we used a cell-permeable fluorescent dye, Hoechst, to rule out the possibility that 5-CF could have generated such a pattern by diffusing through cell membranes. Hoechst freely passes through membranes but does not freely diffuse due to its high affinity for chromatin https://www.thermofisher.com/order/catalog/product/H3570. We therefore opted for feeding it to animals rather than microinjecting a small quantity in the intestine, since it would have probably only stained a few nuclei around the injection site. When we soaked animals into Hoechst, high quantities of the dye penetrated the animal’s intestine and stained all intestinal nuclei, while a vast excess of stain passed through the intestine and from there, stained every nuclei within the animal (Fig. 2*B*). This result indicates that there are no membrane-independent diffusion barriers between the intestine and the other tissues of the animal.

As an additional control, we soaked animals in a saturated 5-CF solution to verify that this dye would not pass through the intestine luminal membrane, which lacks gap junction hemichannels (37). After an hour of soaking, followed by a 30 min recovery period in the absence of dye, animals did not retain any obvious fluorescence apart from within head amphid neurons (Fig. 2*C*), which are open to the environment (38). Overall, we conclude that 5-CF cannot pass directly through cell membranes in *C. elegans*, and based on the observed diffusion pattern after its microinjection in the animal’s intestine (Fig. 1), a significant fraction of it transfers to the germline through an unknown route.

### Small variations in the intestinal microinjection site do not affect 5-CF transfer to the germline

**Figure 3:**
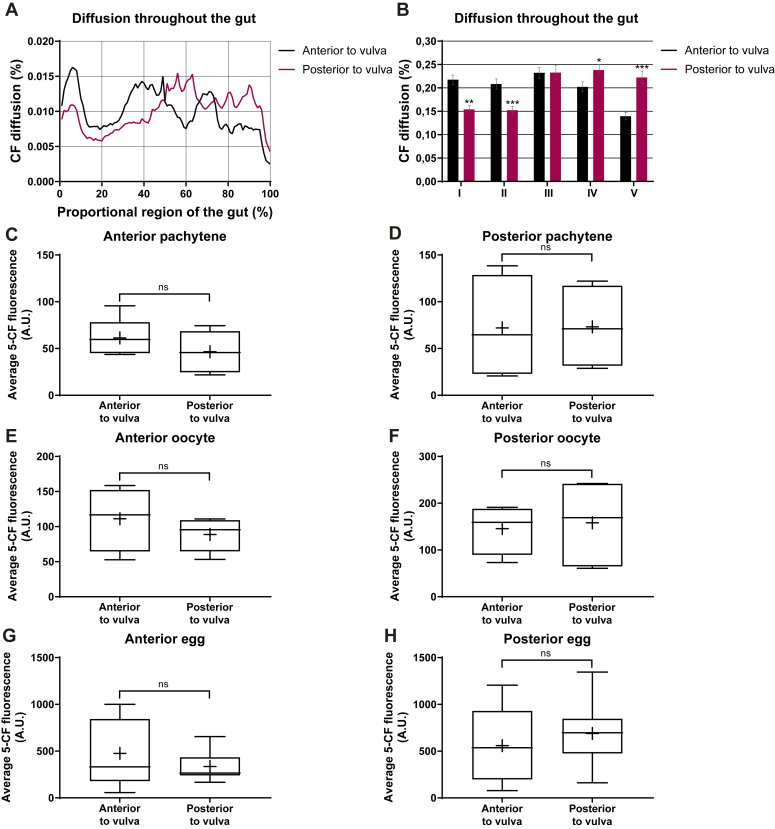
**Small variations in the intestinal microinjection site do not affect 5-CF transfer to the germline.***A*, graphical representation of the 5-CF diffusion across the intestine of WT adults injected either just anterior or just posterior to the vulva (See Experimental procedures). *B*, relative fraction of 5-CF that localized to 5 arbitrary antero-posterior zones of the intestine. Zone I comprises the anterior 20% of the intestine, and zone III, the center portion, encompasses the injection site. *C*–*H*, average 5-CF fluorescence intensities measured in the different germline regions, taking into consideration the injection site. *A*–*H*, sample sizes, *A* and *B*: 7, 9; *C*: 5, 8; *D*: 4, 4; *E*: 4, 5; *F*: 4, 6; *G* and *H*: 5, 10. Statistical tests, B zones I-II and IV-V, *F* and *G*: Mann-Whitney; B zone III, *C*–*E*, *H*: *t* test. 5-CF, 5-carboxyfluorescein.

Before moving into further quantitative comparisons of the 5-CF diffusion pattern, we first verified that small variations in the microinjection site would not dramatically impact the spread of 5-CF from the intestine to the germline. To this end, we injected 5-CF in the intestine of WT adult hermaphrodite, either just anterior or just posterior to the vulva, into the int4/int5 or int5/int6 pair, respectively. After the recovery period, total fluorescence intensity across the entire intestine’s length was measured in each group. The data were doubly normalized to compensate for differences in intestinal length between individuals and in the quantity of dye injected (Fig. 3*A*). For quantification purposes, the intestine was then longitudinally divided into five equal zones, the third zone always encompassing the injection site. There was a slight but significant bias in dye distribution, as animals that were injected just anterior to the vulva, had more dye within the anterior intestinal regions and inversely, the dye preferentially accumulated in the posterior intestine of animals that were injected just posterior to the vulva (Fig. 3*B*).

We then asked whether these small differences in dye distribution within the intestine could influence the transfer of 5-CF from the intestine to the anterior and posterior gonadal arm regions. However, we did not find any significant differences in the transfer of 5-CF to the anterior *versus* posterior germline regions, whether animals were injected in the intestine just anterior or just posterior to the vulva (Fig. 3, *C*–*H*). Overall, these results show that small variations in the intestinal injection site around the vulva, despite that they slightly affect intraintestinal dye distribution, do not significantly impact dye transfer to the germline.

### Transfer of 5-CF to the proximal germline largely requires RME-2-mediated endocytosis

**Figure 4:**
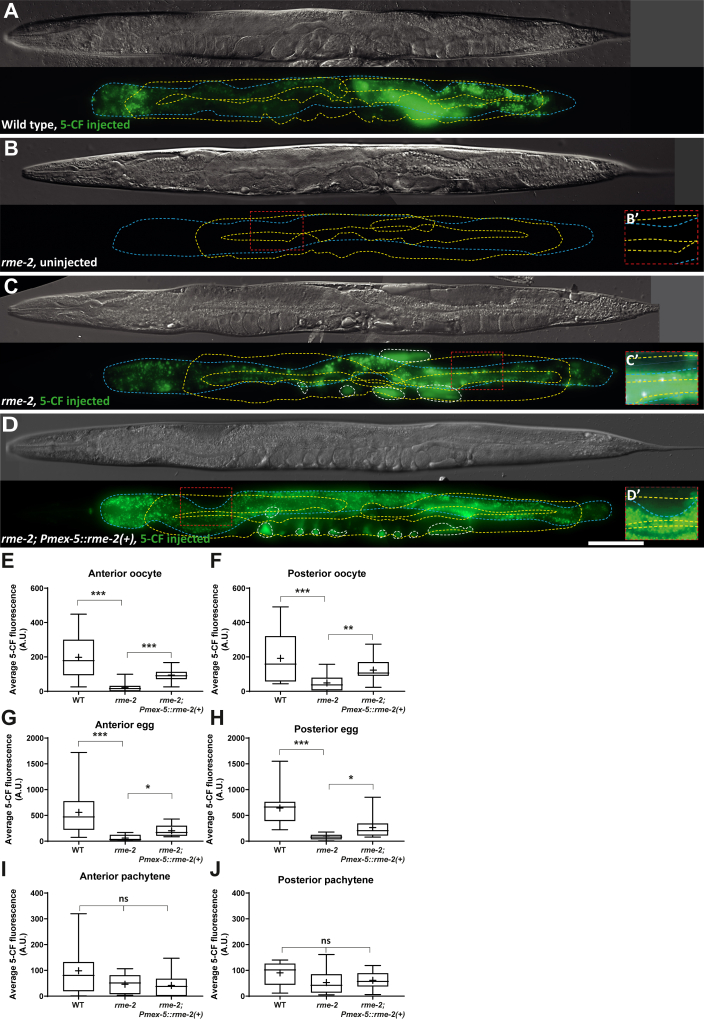
**5-CF transfers from the intestine to the proximal germline mainly through RME-2-mediated endocytosis.***A*–*D*, representative DIC images and fluorescence maximal projections of WT, *rme-2*, and rescued *rme-2* mutant adults that were or were not microinjected with 5-CF in the intestine, near the vulva. The intestine is delimited by a *blue dashed line* while the germline is delimited by a *yellow dashed line*. Where applicable, 5-CF that localized to the pseudocoelom was highlighted by *white dashed lines*. The scale bar represents 100μm. Anterior or posterior germline pachytene regions were enlarged and enhanced in *B’*–*D’* to allow visualization of the dimmer signal present in this area, where applicable. Alleles*, rme-2(b1008)*, *narSi24[Pmex-5::RME-2::WrmScarlet::rme-2 3'UTR]*. *E*–*J*, average 5-CF fluorescence intensities measured in the different regions analyzed of animals of the indicated genotypes. Sample sizes, *E*: 12, 15, 17; *F*: 14, 16, 24; *G*: 21, 15, 16; *H*: 20, 14, 21; *I*: 12, 13, 23, *J*: 7, 13, 10. Statistical tests, *E*–*I*: Kruskall–Wallis; *J*: one-way ANOVA. 5-CF, 5-carboxyfluorescein; DIC, differential interference contrast.

For all intestinal microinjections, we noticed a higher concentration of 5-CF transferring into the oocytes and eggs relative to the pachytene region (Fig. 1). Several factors could explain this phenomenon, including the presence of distal-to-proximal cytoplasmic streaming within the gonad arms (39), or potential differences in gap junction abundance, size and/or in gating regulation between these regions. However, the exo/endocytosis pathway is known to transport yolk proteins from the intestine to the maturing oocytes (28). Indeed, yolk proteins are synthesized in the intestine, secreted into the pseudocoelom and endocytosed by oocytes through the yolk receptor RME-2, an low-density lipoprotein receptor-like molecule expressed by oocytes (28). We therefore asked whether a fraction of the 5-CF that transferred from the intestine to the oocytes, traveled along with the yolk proteins. To evaluate this potential contribution, we microinjected 5-CF in the intestine of *rme-2(b1008)* mutants and monitored its transfer to the germline. We observed a drastic reduction in the transfer of 5-CF from the intestine to the oocytes and distal eggs in *rme-2(b1008)* mutants, meaning that the major fraction of 5-CF uses this exo/endocytic route to travel from the intestine to the proximal germline (Fig. 4, *A*–*H*). However, comparable 5-CF concentrations reached the germline pachytene region in WT and *rme-2(b1008)* mutants (Fig. 4, *I* and *J*). We also note that a small fraction of 5-CF, comparable to the fraction that transferred to the pachytene region in WT or *rme-2(b1008)* mutants, still transferred to the oocytes and eggs in *rme-2(b1008)* mutants (Fig. 4, *E*–*H*). Moreover, consistent with a reduced dye uptake by the germline in these mutants, more of the dye remained in their intestine (Fig. S2), while some also visibly highlighted their pseudocoelomic fluids (Fig. 4*C*). Altogether, we conclude that in the WT, a large proportion of 5-CF transfers from the intestine to the oocytes *via* exo/endocytosis and the RME-2 yolk receptor, but that a smaller yet significant part gets into the germline through another route.

To confirm the RME-2 requirement for dye transfer, we used CRISPR/Cas9 to reintroduce a single-copy transgene expressing *RME-2(+)::WrmScarlet*, as driven by the germline-specific *mex-5* promoter, in *rme-2(b1008)* mutants (40, 41). This transgene significantly rescued *rme-2(b1008)* defects, including its low brood size, embryonic, and larval lethality, in addition to restoring 5-CF transfer to oocytes and eggs (Figs. 4, *C*–*H*, S2).

### Sheath cells do not overly accumulate 5-CF in the absence of gap junction connections to the germline

Despite the relatively low levels of 5-CF that we measured in the proximal gonadal sheath cells (Figs. 1, S1), to further probe the potential involvement of the gap junctions that connect them to the germline, we microinjected 5-CF in the intestine of *inx-8 inx-9* double mutants. These animals have very few and often necrotic germ cells as the soma-to-germline gap junctions *inx-8* and *inx-9* encode are required for germ cell proliferation (18). Since the sheath cells were crumpled together around the few, when any, germ cells, it was not possible to distinguish whether the relatively faint fluorescence that we detected there was coming from the germ or sheath cells (Fig. S3). Nevertheless, the 5-CF levels detected in the anterior and posterior sheath/germ cells of *inx-8 inx-9* mutants was not different from what we had measured within the anterior and posterior pachytene region of WT animals (Fig. S3). As with *rme-2* mutants, a greater proportion of the 5-CF remained in the intestine of *inx-8 inx-9* mutants than within that of WT animals, while the dye also visibly highlighted their pseudocoelomic fluids (Fig. S3), which is consistent with 5-CF not being endocytosed by the inexistent oocytes of these mutants. We conclude that in the absence of soma-to-germline gap junctions, the gonadal sheath cells do not accumulate more 5-CF, which instead remains largely trapped in the intestine and pseudocoelom.

### 5-CF also transfers from the germline to the intestine

**Figure 5:**
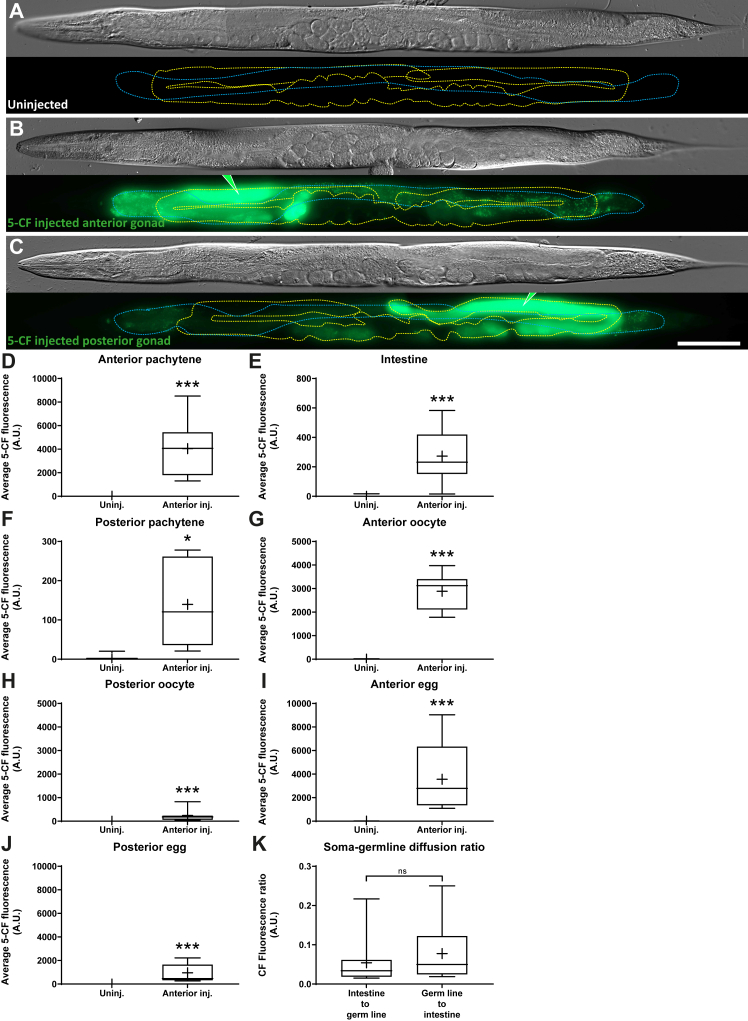
**A small membrane-impermeable molecule transfers from the germline to the intestine.***A*–*C*, representative DIC images and fluorescence maximal projections of WT adults that were or were not microinjected with 5-CF in the pachytene region of the anterior or posterior gonad arm (green pipette tip). The intestine is delimited by a *blue dashed line* while the germline is delimited by a *yellow dashed line*. The scale bar represents 100μm. *D*–*J*, average 5-CF fluorescence intensities measured in the different regions analyzed in animals microinjected with 5-CF in the anterior pachytene region. *K*, average 5-CF fluorescence intensity transfer ratios between the intestine and the germline pachytene region, depending on the injection site. *D*–*K*, Sample sizes, *D*: 7, 11; *E*: 9, 11; *F*: 6, 6; *G*: 9, 8; *H*: 8, 7; *I*: 8, 11; *J*: 7, 9; *K*: 13, 10. Statistical tests, *D*–*G*: Welch’s; *H*–*K*: Mann-Whitney. 5-CF, 5-carboxyfluorescein; DIC, differential interference contrast.

Next, we carried out the reverse experiment and asked whether 5-CF microinjected in the germline pachytene region would remain within that gonad arm, or transfer to the intestine and/or other tissues of the animal. Such “retrograde” transfer was not necessarily expected as for example, the yolk exo/endocytosis route is widely viewed as being unidirectional (28, 42, 43). Yet, when we microinjected 5-CF in the anterior gonad arm’s pachytene region, the dye transferred into the intestine and visibly spread over its whole length (Fig. 5, *A* and *B*). Quite strikingly, the dye was also detectable in the posterior gonad arm’s pachytene region, proximal-most oocytes, and distal-most eggs (Fig. 5, *A*, *B*, *D*–*J*). The same phenomenon occurred following injection in the posterior arm, although we did not inject enough animals to perform quantifications (Fig. 5*C*). Since there were no apparent dyes in the uterus, we suspect that the dye transferred from the injected gonad arm to the pseudocoelom and intestine, and from here and there, to the uninjected gonad arm.

We noticed that for both intestinal and distal germline microinjections the dye fluorescence intensity did not equalize between these two tissues or regions, despite allowing ample time to diffuse, and remained more concentrated near the injection site (Figs. 1 and 5). By measuring the fluorescence transfer ratios, we found that similar relative fluorescence intensities were attained in the distant site, regardless of whether the dye was injected in the intestine or germline pachytene (Fig. 5*K*). For intestine to distal germline and distal germline to intestine, the transfer fluorescence intensity ratio’s 95% confidence intervals were 0.019 to 0.090% and 0.024 to 0.13%, respectively. Altogether, these results establish the possibility of a bidirectional small membrane-impermeable molecule transfer between the *C. elegans* intestine and germline. The results further imply the existence of a bidirectional partial diffusion barrier slowing down 5-CF transfer between the intestine and distal germline, since fluorescence intensity at the distant site was lower than at the injected site. That barrier however does not apply to the *rme-2*-dependent transfer from the intestine to the firsts ovulated oocytes (eggs), where 5-CF concentrations did equalize (Fig. 1*R*).

### Intestine to distal germline 5-CF transfer efficiency does not vary together with GSC proliferation rates

**Figure 6:**
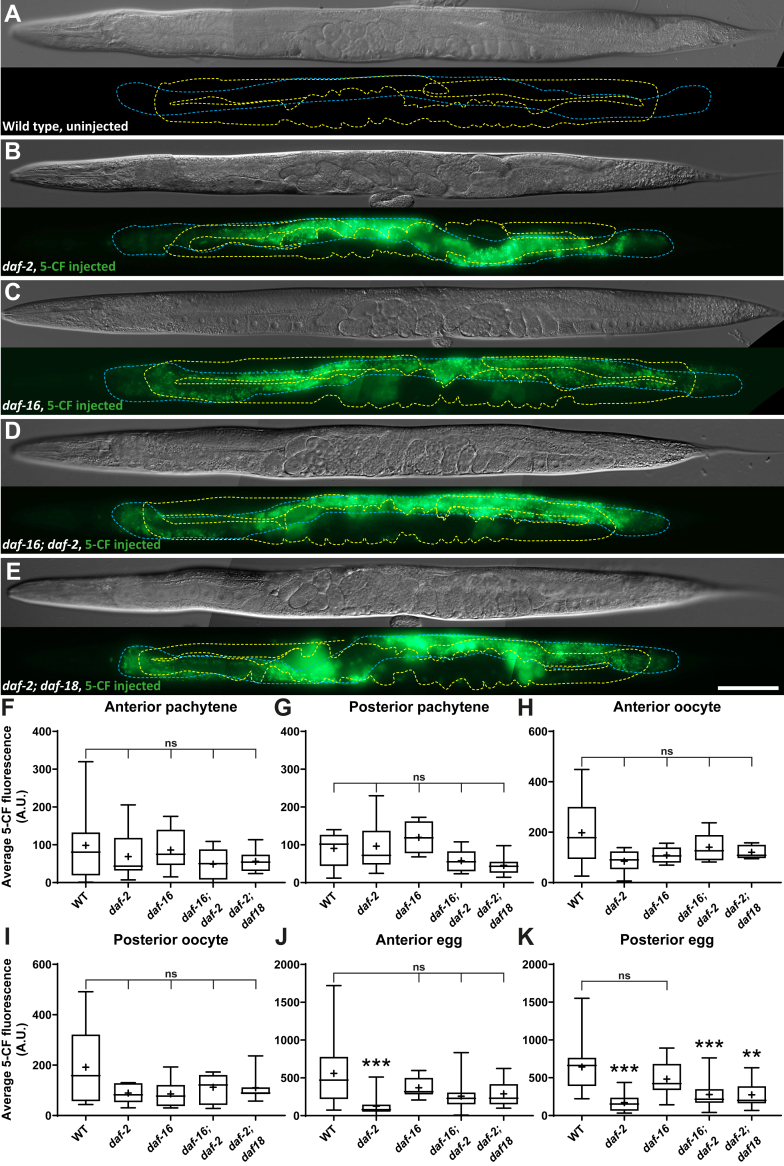
**Reduced IIS does not affect intestine to distal germline 5-CF transfer.***A*–*E*, representative DIC images and fluorescence maximal projections of WT and IIS mutant adults that were or were not microinjected with 5-CF in the intestine near the vulva. The intestine is delimited by a *blue dashed line* while the germline is delimited by a *yellow dashed line*. The scale bar represents 100μm. *F*–*K*, average 5-CF fluorescence intensities measured in the different regions analyzed. Alleles, *daf-16(mu86)*, *daf-2(e1370)*, *daf-18(ok480)*. Sample sizes, *F*: 12, 12, 16, 21, 10; *G*: 7, 15, 4, 9, 8; *H*: 12, 12, 5, 9, 5; *I*: 14, 11, 6, 15, 7; *J*: 21, 23, 16, 22, 13; *K*: 20, 22, 17, 21, 13. Statistical tests, *F*, *I*–*K*: Kruskal-Wallis; *G*: one-way ANOVA; *H*: Brown-forsythe and Welch’s ANOVA. 5-CF, 5-carboxyfluorescein; DIC, differential interference contrast; IIS, insulin/IGF-1 signaling.

To determine whether the modulation of small molecule transfer from the intestine to the germline could underlie the regulation of GSC proliferation rates, we microinjected 5-CF in the intestine of different mutants in which GSC proliferation is known to be reduced. We first monitored 5-CF diffusion in insulin-like receptor *daf-2* mutants in which GSC proliferation is reduced due to lack of growth factor stimulation downstream of nutrient uptake (20, 21, 22). This reduction in proliferation requires the activity of both DAF-18/PTEN and DAF-16/FOXO (20, 21, 22). We did not detect any differences in the efficiency of 5-CF transfer from the intestine to the germline pachytene region and oocytes between any of the tested mutant combinations in this pathway (Fig. 6, *A*–*I*). There was however a significant reduction of 5-CF transfer from the intestine to the eggs (former oocytes) in *daf-2* mutants (Fig. 6, *J* and *K*). This is likely linked to the slower rates of oocyte maturation and reduced vitellogenesis of these mutants (44, 45), which is the main route of dye transfer from the intestine to the oocytes (Fig. 4). Because similar proportions of 5-CF still transferred from the intestine to the distal germline in these *daf-2* mutants that have reduced GSC proliferation, we infer that this reduction in proliferation does not involve the instauration of any further restrictions to the transfer of small membrane-impermeable molecules to the distal germline, such as one that could have been imposed by soma-to-germline gap junction closure.

**Figure 7:**
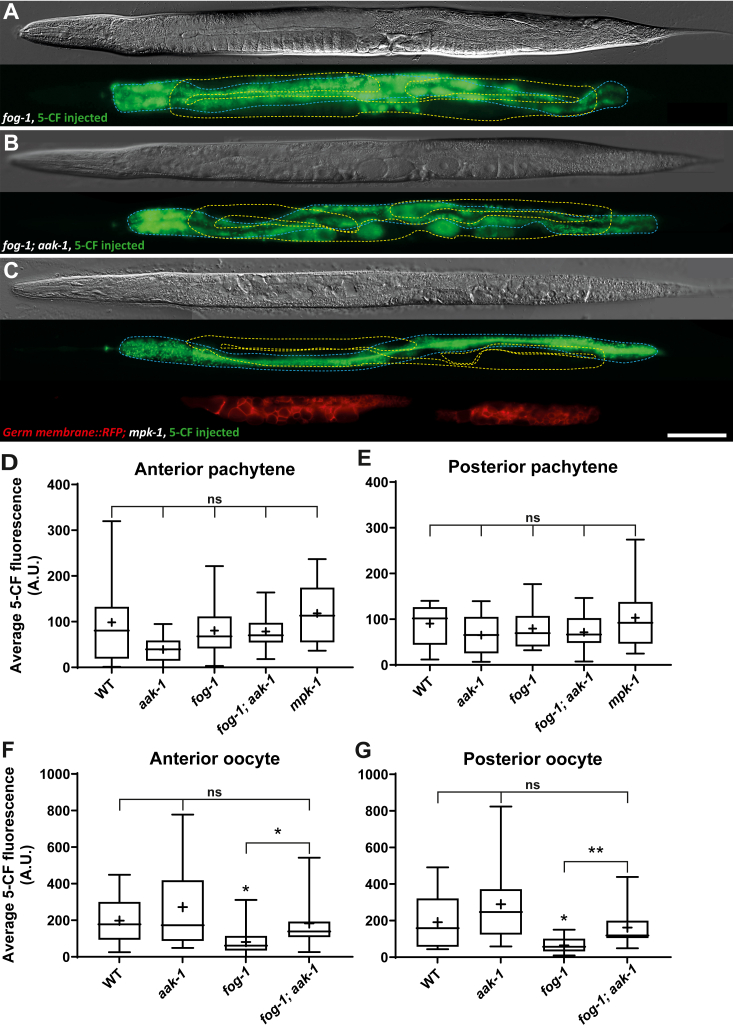
**Reduced MPK-1 signaling does not affect intestine to distal germline 5-CF transfer.***A*–*C*, representative DIC images and fluorescence maximal projections of *fog-1*, *fog-1; aak-1*, and *mpk-1* mutant adults that were microinjected with 5-CF in the intestine near the vulva. The intestine is delimited by a *blue dashed line* while the germline is delimited by a *yellow dashed line*. The scale bar represents 100μm. A germ membrane labeling RFP was introduced in *C* to facilitate analysis. *D*–*G*, average 5-CF fluorescence intensities measured in the different regions analyzed in the indicated genotypes. Alleles, *fog-1(q785)*, *mpk-1(ga117)*, *aak-1(tm1944)*, *cpSi20[Pmex-5::TagRFP-T::PH::tbb-2 3'UTR + unc-119(+)]*. Sample sizes, *D*: 12, 25, 10, 15, 12; *E*: 7, 11, 8, 14, 12; *F*: 12, 27, 18, 18; *G*: 14, 21, 18, 18. Statistical tests, *D*: Brown-Forsythe and Welch’s ANOVA; *E*: one-way ANOVA; *F*–*G*: Kruskal–Wallis. 5-CF, 5-carboxyfluorescein; DIC, differential interference contrast.

In parallel to IIS, somatic MPK-1 activity cell nonautonomously promotes GSC proliferation (25, 46). MPK-1 signaling is however reduced in germline-feminized *fog-1(ø)* mutants, which lack sperm and have reduced oocyte needs, through a homeostatic feedback loop requiring AAK-1/AMPK activity (22, 23, 47, 48, 49). We therefore asked whether the reduced GSC proliferation rates of *fog-1(ø)* and *mpk-1(ø)* mutants could be associated with a restriction in the transfer of small membrane-impermeable molecules from the intestine to the distal germline. Yet, again we did not detect any differences in the transfer of 5-CF from the intestine to the germline pachytene area between any of the tested genotypes and the WT (Fig. 7, *A*–*E*). However, there was a significant reduction of 5-CF transfer from the intestine to the oocytes in *fog-1(ø)* mutants, which was suppressed by *aak-1(ø)* (Fig. 7, *F* and *G*). This may reflect a reduction in yolk transport when sperm is depleted, and oocytes arrest and accumulate (50), as dye transfer reverted to normal when the *aak-1(ø)* mutation was added to prevent oocyte arrest and accumulation (23). We therefore conclude that the downregulation of GSC proliferation that occurs when *mpk-1* signaling is reduced does not involve any significant restriction in the efficiency of 5-CF transfer from the intestine to the distal germline. Overall, the reduced GSC proliferation rates that characterize *daf-2*, *fog-1(ø)* and *mpk-1(ø)* mutants cannot be the result of changes in the intestine to distal germline transfer efficiency of any small membrane-impermeable molecule that would behave like 5-CF. In all three mutants, however, reduced GSC proliferation may be linked to reduced yolk/5-CF endocytosis by proximal oocytes, although this relationship is unlikely to be direct.

## Discussion

In this study, we investigated the distribution of the small membrane-impermeable green fluorescent dye 5-CF following its microinjection in the *C. elegans* intestine and germline. We found that intestine-injected 5-CF diffused away from injected cells and spread through the whole intestine. A fraction of the dye also transferred from the intestine to the germline, while we did not observe any large accumulation elsewhere in the animal. The larger part of the intestine to germline dye transfer occurred *via* the exo/endocytosis route, requiring the RME-2 yolk receptor. As such, intestine-injected 5-CF abundantly labeled the proximal-most oocytes, some of which had turned into eggs during the recovery period. However, significant 5-CF amounts also transferred more globally to the germline, independently of RME-2, and thus must have used another route. 5-CF being membrane-impermeable, but small enough to pass through gap junctions, this route could have included the gap junctions that link the somatic gonad to the germline. However, relatively little 5-CF transferred to the gonadal sheath cells in either the WT or *inx-8 inx-9* double mutant that lack soma-to-germline gap junctions. Instead, the dye visibly labeled the pseudocoelom, in both the *inx-8 inx-9* and *rme-2(b1008)* backgrounds, suggesting that it can accumulate there when it is not uptaken by maturing oocytes, not within the gonadal sheath cells. Thus, it appears unlikely that the dye traveled through the somatic gonad and soma-to-germline gap junctions to transfer from the intestine to the germline. We favor the hypothesis that the dye was secreted into the pseudocoelom by the intestine, and was uptaken from there directly by the germline, but also to some extent, by other endocytic tissues such as the coelomocytes and hypodermis. The small quantities of dye that were detected in the gonadal sheath cells could have resulted from a direct uptake from the pseudocoelom, and/or as a result of diffusion from the germline through gap junctions. However, as 5-CF concentrations within proximal sheath cells were below that within the neighboring oocytes and eggs, the dye did not freely diffuse across the gap junctions that connect these two tissues. This imbalance in dye concentration on each side of these soma-to-germline gap junctions suggests that the dye might have an affinity for molecules that are abundant in oocytes but excluded from the sheath cells, such as yolk components (further discussed below). In any cases, the way by which the *rme-2* independent germline uptake occurs remains unclear.

Several controls were performed to strengthen this paradox-creating conclusion. We microinjected GFP, a molecule that is too large to pass through gap junctions. As expected, GFP could not escape from the cells that were targeted by the injection, indicating that there are no membrane-free gaps between the intestine and the germline. Next, we fed animals Hoechst, a cell-permeable fluorescent dye. Here, the dye did diffuse from the intestine to the germline, while the dye also transferred to all the other tissues of the animal. This revealed that there were no membrane-independent diffusion barriers in the animal, and that cell membranes do participate in the apparent biases in 5-CF transfer. Then, we soaked animals in a saturated 5-CF solution to confirm that this dye could not pass through membranes that lack gap junctions, such as the intestine luminal membrane. Finally, and perhaps most convincingly, we microinjected the dye in the germline pachytene region, in either gonad arm, and showed that it transferred in the other direction, and accumulated in these animal’s intestine. Our results therefore establish strong grounds for the existence of bidirectional small membrane-impermeable molecule mediated soma-germline interactions. As such, this intestine-germline axis of signaling may not only serve to promote germline functions such as GSC proliferation and oocyte maturation/ovulation (12, 18, 25), but perhaps also influence somatic outcomes such as aging, in response to germline cues. For example, germline ablation is well-known to considerably extend lifespan (51). Small life shortening membrane-impermeable molecules could therefore emanate from the growing germline and directly affect the animal’s intestine, which is largely implicated in lifespan regulation (52, 53).

Previous work has uncovered a couple of long-distance intestine-to-germline interactions in *C. elegans*. In one example, cytidine deaminases were found to be required in the gut or somatic gonad of the animal to nonautonomously promote GSC proliferation. In this case, it was concluded that low uridine/thymidine (these enzymes products) in the gut and somatic gonad suppressed GLP-1/Notch signaling and reduced GSC proliferation (54). In the second example, we found that MPK-1/ERK activity was required, again either in the animal’s intestine or somatic gonad, to promote GSC proliferation (25). This current piece now demonstrates that small membrane-impermeable building blocks, such as uridine and thymidine, may effectively transfer from the intestine to the germline. This legitimizes the hypothesis that cytidine deaminases and MPK-1 may promote germline proliferation *via* the production of small metabolites in either the intestine or the somatic gonad; independently of the tissue in which they are produced, since the metabolites can transfer from either tissue to the germline to promote proliferation. As such, the intestine and germ tissues may share many small membrane-impermeable molecules such as ions, second messengers, and small metabolites implicated in a number of biological processes.

Upon injection of 5-CF in the *C. elegans* intestine, and despite some small biases linked to the microinjection site, we noticed that the dye concentration roughly equalized throughout the length of the gut. This was expected due to the presence of gap junctions coupling all cells together within this tissue (55). We noticed that a fraction of the dye reached the germline, a large part of which used the RME-2-dependent exo/endocytosis route, the passageway that conveys the yolk proteins from the intestine to the oocytes, to reach the proximal germline. The exact route used by the remaining smaller fraction of the dye that traveled from the intestine to the germline however remains obscure. Yet at least one of these routes must allow bidirectional exchanges as significant quantities of the dye also transferred from the germline to the intestine. The intestine is separated from the somatic gonad and germline by a space containing interstitial fluid: the pseudocoelom (55). Interestingly, when GFP is secreted into the pseudocoelom from any tissue, it is predominantly taken up by the animal’s six coelomocytes, scavenger cells that nonspecifically endocytose pseudocoelomic fluid (56, 57, 58). In a pioneering piece Fares and Greenwald microinjected several other dyes in the pseudocoelomic space to investigate endocytosis by the animal’s coelomocytes. Interestingly, among the small molecules tested, only the dyes that appeared to adhere to yolk granules were not efficiently endocytosed by the coelomocytes, and ended up in the oocytes (58). Combining our results with these early observations, it is likely that 5-CF adhered to the yolk granules and was secreted from the intestine into the pseudocoelom *via* exocytosis along with the yolk. This would explain why a large fraction of it was endocytosed by oocytes *via* their RME-2 receptors. If 5-CF indeed adheres to yolk granules, another yet to be identified, and more widely expressed but less prominent, yolk receptor could then be responsible for the uptake of the remaining fraction of the dye throughout the germline. Yet, this would still not explain the transfer that occurred in the other direction, from the germline to the intestine, as yolk transport is believed to occur unidirectionally from the intestine to the germline (28, 42, 43). Interestingly, our *rme-2* rescuing transgene was unexpectedly expressed in the intestine, despite the fact we used a germline-specific promoter (Fig. S3). Consistent with this, we discovered up to three putative GATA motifs within its intronic regions that could be responsible for driving intestinal expression (59). If endogenous RME-2 was present at the surface of the intestine, it could explain the uptake/reuptake of the yolk (and 5-CF) from the pseudocoelom. How the germline would secrete it is also unclear. As for the RME-2-independent transfer of small membrane-impermeable molecules, several alternative routes are possible, such as those implicated in the intertissue transfer of RNA molecules (60, 61) or nucleosides (62). Finally, 5-CF could also mix in with the range of small molecules that are packed into the microvesicles and exosomes that are released by many cell types, including the *C. elegans* intestine (63). Deeper investigations will however be required to identify the exact mechanism(s) underlying the reciprocal and RME-2-independent exchange of small membrane-impermeable molecules between the intestine and germline.

We finally aimed to analyze the distribution of 5-CF across different genotypes. Before conducting this analysis, we verified that small variations in the microinjection site did not widely affect the transfer of 5-CF from the intestine to the germline. Although the dye preferentially accumulated within the anterior half of the intestine in animals injected just anterior to the vulva and in the posterior half when injected just posterior to the vulva, this small difference in dye distribution within the intestine did not significantly impact transfer of 5-CF to the germline. As such, we could investigate whether restrictions in the intestine-to-germline transfer of small membrane-impermeable molecules could underlie the downregulation of GSC proliferation rates that occurs in insulin/IGF-1 and MPK-1 pathway mutants. We found that the efficiency of 5-CF transfer from the intestine to the germline pachytene region remained stable across all tested genetic backgrounds, regardless of their published rates of GSC proliferation. 5-CF transfer to the firsts oocytes (eggs) was however reduced in both *daf-2* and *fog-1* mutants (Figs. 6 and 7). Consistent with these results, *daf-2* mutants were reported to have reduced vitellogenesis and yolk transport from intestine to oocytes (45, 64, 65). Reduced IIS however did not lower the efficiency of 5-CF transfer to the distal part of the germline, suggesting that it downregulates GSC proliferation without influencing the transfer of 5-CF-like small membrane-impermeable molecules to this part of the germline. Similarly, the inactivation of MPK-1/ERK signaling, either through a null *mpk-1* mutation or through its homeostatic downregulation by germline feminization (*fog-1* background), did not modify the pattern of 5-CF transfer from the intestine to the distal germline (we could not test diffusion to the oocytes in *mpk-1(ø)* as their germ cells arrest at the late pachytene stage (46)). Together, our results establish that if MPK-1 activity in the intestine or somatic gonad generates small membrane-impermeable molecules, these could transfer from either of these tissues to the germline, where they could promote GSC proliferation. At the same time, our work also represses the idea that regulation of GSC proliferation rates could involve small 5-CF-like molecule transfer control, including by gap junction gating.

Soma-germline interactions occur in many other organisms and the disruption of these interactions perturbs several aspects of germ cell development and function, which can have significant implications on reproductive success, but also on the health and survival of the organism (15, 18, 66, 67). Unraveling the molecular mechanisms underlying soma-germline interactions may therefore lead to the development of new therapeutic strategies related to germline dysfunction, but perhaps also toward healthy aging. More broadly, the transfer of small membrane-impermeable molecules across tissues may be implicated in a range of developmental and intercellular signaling processes, and thus deregulation could underlie several pathologies, including those implicating stem cell proliferation defects, such as many degenerative diseases and cancers.

## Experimental procedures

### Strains

Animals were maintained on standard nematode growth medium plates and fed *Escherichia coli* bacteria of the stain OP50 (68). The Bristol isolate (N2) was used as WT throughout. Prior to all assays, animals were grown at 15 °C, and based on vulva development (69), were transferred at the late L4-stage to a new plate at 25 °C for an additional 24 h before any subsequent treatment. We use “A1” to refer to these day-1 adults (22). The following alleles and transgenes were used. LGI: *fog-1(q785)*, *daf-16(mu86)*, *narSi24[Pmex-5::RME-2::WrmScarlet::rme-2 3'UTR]*, *foxSi41[Pdpy-7::tomm-20::mKate2::HA::tbb-2 3' UTR]* (70). LGII: *cpSi20[Pmex-5::TagRFP-T::PH::tbb-2 3'UTR + unc-119(+)]*. LGIII: *daf-2(e1370)*, *mpk-1(ga117)*, *aak-1(tm1944)*, *unc-119(ed3)*. LGIV: *daf-18(nr2037)*, *rme-2(b1008)*, *inx-8(tn1474)*, *inx-9(ok1502)*. Extrachromosomal arrays: *narEx110[Plim-7::TagBFP2; Pmyo-2::GFP], narEx113[coel::RFP; unc-119(+)]*, *narEx114[coel::RFP; unc-119(+)]*, *tnEx195[inx-8(+); inx-9(+); Psur-5::GFP]*. The following rearrangements were used. *hT2[bli-4(e937) let-?(q782) qIs48]* (I;III), *qC1[dpy-19(e1259) glp-1(q339) qIs26[rol-6(su1006)gf; Plag-2::GFP]]* III.

### Transgenics

For the germline rescue of *rme-2(b1008)* we used the Gibson method (71) to modify pCC361 (72) into a *Pmex-5::RME-2::WrmScarlet::rme-2 3'UTR* CRISPR/Cas9 repair template plasmid (pXA11) designed for single-copy insertion targeting the ttTi4348 locus on LG I (40, 41). We used the WT genomic *rme-2* sequence along with 857 base pairs from its 3’UTR. Three independent insertions were obtained in the WT background, and after excising the selection cassette, we crossed one (*narSi24*) into the *rme-2(b1008)* background.

For the blue fluorescent sheath cell reporter, we used the Gibson method (71) to generate a *Plim-7::TagBFP2* plasmid (pLDY14) using the *Plim-7* sequence from pGC197 (73) and *TagBFP2* sequence from pJJR81 (Addgene #75029), and microinjected it (50 ng/μL) together with pMR352 *[Pmyo-2::GFP]* (74) (5 ng/μL) and pKSII (95 ng/μL) to generate *narEx110[Plim-7::TagBFP2; Pmyo-2::GFP]* (32).

To mark the coelomocytes with red fluorescence, we microinjected a *coel::RFP* (50 ng/μL) plasmid (Addgene #8938) together with MM051[unc-119(+)] (75) (50 ng/μL each) and pKSII (150 ng/μL) in *unc-119(ed3)* mutants. We used two arrays for analysis: *narEx113* and *114*.

### Microinjection

A1 hermaphrodites were microinjected either in the intestine (dorsally, either just anterior or just posterior to the vulva) or distal gonad (pachytene region) with a small volume (estimated at 30-50 pl) (32) of a 1x PBS solution saturated with 5-CF (MilliporeSigma #86826) or 1mg/ml GFP (Abcam #ab84191), using techniques previously described (32). Briefly, animals were picked into a drop of halocarbon oil and immobilized by sticking them against a dried agarose pad on a cover slip. We used an Eppendorf InjectMan 4 micromanipulator coupled to an inverted Zeiss Axiovert A1 equipped with a 63x dry objective (NA 0.85) for all microinjections. Animals never stayed more than 2 minutes on the injection pad, and after the injection, they were allowed to recover on a seeded plate for 1 hour at room temperature. This time easily allowed the 5-CF to diffuse throughout length of the animal (Fig. 1*B*). Animals that did not entirely recover from the microinjection procedure were discarded.

### Stainings

A1 hermaphrodites were harvested from plates in 1 ml of M9 buffer. The suspension was pipetted into a 1.5ml microtube and centrifuged at 2500 rpm for 1 min. The supernatant was removed to leave worms in about 20 μl of M9 buffer. Subsequently, 50 μl of either a saturated 5-CF or of 18mM Hoescht (Thermo Fisher Scientific, H3570) was mixed into the microtube. Animals were stained on a shaker plate at 80 rpm for 1 h at room temperature in the dark. After the incubation time, animals were pipetted on a seeded plate for 30 min to recover and allow the complete clearing of the stain from their intestinal lumen, before they were imaged.

### Image acquisition

For imaging, A1 animals were paralyzed by soaking in a 0.1% Tetramisole (Sigma-Aldrich, L9756) M9 solution for 5 minutes and mounted on a 3% agarose pad. Differential interference contrast (DIC) and epifluorescence images were acquired every micron using a Plan-Apochromat 20x dry objective (NA 0.8) mounted on an inverted Zeiss Axio Observer.Z1. Images were stitched using the Zen 2.6 software (https://www.zeiss.com/microscopy/en/products/software/zeiss-zen.html) and, for display purposes, animals were computationally straightened using ImageJ (https://imagej.net/ij/). All DIC images show single focal planes, while fluorescence images show maximal projections that were thresholded to remove the relatively faint background autofluorescence. The DIC-fluorescence overlay images in Figure 1, *K*–*Q* however, were realized with single fluorescent focal planes to represent how the analysis was realized. All fluorescent images in Figs. S1–S3 also show single focal planes. The exact same acquisition parameters were used for all samples within each assay but could vary between separate assays.

### Fluorescence quantification

Fluorescence quantification was performed using ImageJ. To quantify 5-CF diffusion between the intestine and the different germline regions, or from the germline pachytene region to the intestine, we first measured the whole animal’s total fluorescence intensity for each subject. We created a Z-project using the sum density function, then selected a region of interest (ROI) corresponding to the whole animal and measured its integrated density. We used the average value for all WT injections to normalize the fluorescence intensities obtained for the other genotypes, and for individuals within each genotype, in order to compensate for variations in the quantity of fluorochrome injected. We then placed up to three (when possible) identical circular ROIs of 99,451 μm^2^ and averaged their fluorescence intensities in the focal plane where the middle of the following tissues was in focus: intestine, anterior and posterior germline pachytene regions, proximal-most (−1) oocytes, distal-most (+1) eggs, or the sheath/germ cells in *inx-8 inx-9* doubles. For coelomocytes, we placed up to three circular ROIs of 8 μm^2^ per coelomocyte, in up to six coelomocytes per individual, and averaged their fluorescence intensities. For the lateral hypodermis, we used the average of two ≈ 100 μm long by five pixels wide linear ROIs placed in the anterior and posterior half of the tissue. For the anterior and posterior sheath cells in WT animals, a ≈ 50 μm long by two pixels wide linear ROI was traced in the tissue adjacent to the −1 and −2 oocyte. Fluorescence intensity values for each region were normalized based on the total fluorescence intensity in the whole animal and were background subtracted to remove the signal coming from natural autofluorescence using the respective regions of uninjected controls of the same genotype. For all fluorescence intensity graphs except Figure 1*C*, this average endogenous fluorescence was subtracted from all samples, including uninjected controls, such that the fluorescence intensity values for injected animals are directly proportional to fluorophore concentrations. Whenever sheath cells were labeled with BFP2, due to the presence of *Pmyo-2::GFP* expression in the pharynx, we digitally cut off their heads before evaluating whole animal fluorescence.

For the quantification of 5-CF along the length of the intestine in Figure 3, *A* and *B*, an 80-pixel wide segmented line was manually drawn over the length of the intestine and selected as the ROI. Average pixels intensities at each *x*-coordinate within the ROI were obtained using the Plot Profile tool. To normalize for intestine length, each *x*-coordinate from the plot profile line was divided by the length of the line and all pixel intensities were pooled by integer from 1 to 100 at a 0,01 span. We then used uninjected controls to subtract the background autofluorescence from all images. To normalize for variations in microinjection volumes, all pixel intensity values (y-coordinates) were divided by the animal’s intestine length normalized total pixel intensity. Finally, the adjusted (length-normalized, background subtracted, and intensity-normalized) fluorescence intensities were averaged for each group. For Figure 3, *A* and *B*, the intestine was longitudinally divided into five equal zones, and all y-coordinates were averaged within each zone for Figure 3*B*. The third zone always comprised the injection site.

### Brood size, embryonic, and larval lethality

For fecundity, according to standard procedures (76), late L4 larvae were singled at 15 °C. Animals were transferred to a new plate every 24 h, after which the number of eggs laid and hatched larvae were scored. The assay was stopped when an individual laid no eggs over 24 h. For embryonic lethality, the number of unhatched eggs after 24 h was recorded (2x checked after 48 h). For larval lethality, the number of dead or arrested larvae was scored 5 days later.

### Statistical analyses

Statistical analyses were performed using GraphPad Prism 10.3.1 (https://www.graphpad.com/features). Each dataset’s normality was verified using the Shapiro-Wilk test. Variance was verified using the F test for pairwise comparisons, or the Brown–Forsythe test when comparing multiple samples. *p* values lower than 0.05, marked with one asterisk (∗), were considered as statistically significant against the control unless otherwise indicated by brackets; two asterisks: *p* < 0.01; three asterisks: *p* < 0.001. Sample sizes are always indicated from left to right. Statistical tests are indicated in the figure legends and were chosen according to the following criteria.

For pairwise comparisons with normal distributions and equal variances, we used the unpaired two-tailed *t* test. When the distribution was not Gaussian, we used the Mann-Whitney test. When the distribution was normal, but the variance was unequal, we used Welch’s *t* test.

To compare multiple samples having normal distributions and equal variances, we used the one-way ANOVA with Tukey’s multiple comparisons. When the distribution was not Gaussian, we used the Kruskal–Wallis with Dunn’s multiple comparisons. When the distribution was normal, but the variance was unequal, we used the Brown–Forsythe and Welch’s ANOVA with Dunnett’s T3 multiple comparisons.

## Data availability

Data are available upon request (patrick.narbonne@uqtr.ca).

## Supporting information

This article contains supporting information.

## Conflict of interest

The authors declare that they have no conflicts of interest with the contents of this article.
