# Supplementary material for: Bidirectional transfer of a small membrane-impermeable molecule between the Caenorhabditis elegans intestine and germline
Source: J Biol Chem. 2024 Nov 5;300(12):107963. doi: 10.1016/j.jbc.2024.107963 (PMC11647508; doi:10.1016/j.jbc.2024.107963)
Supplement: Supporting Information [file mmc1.pdf]

Supporting Information

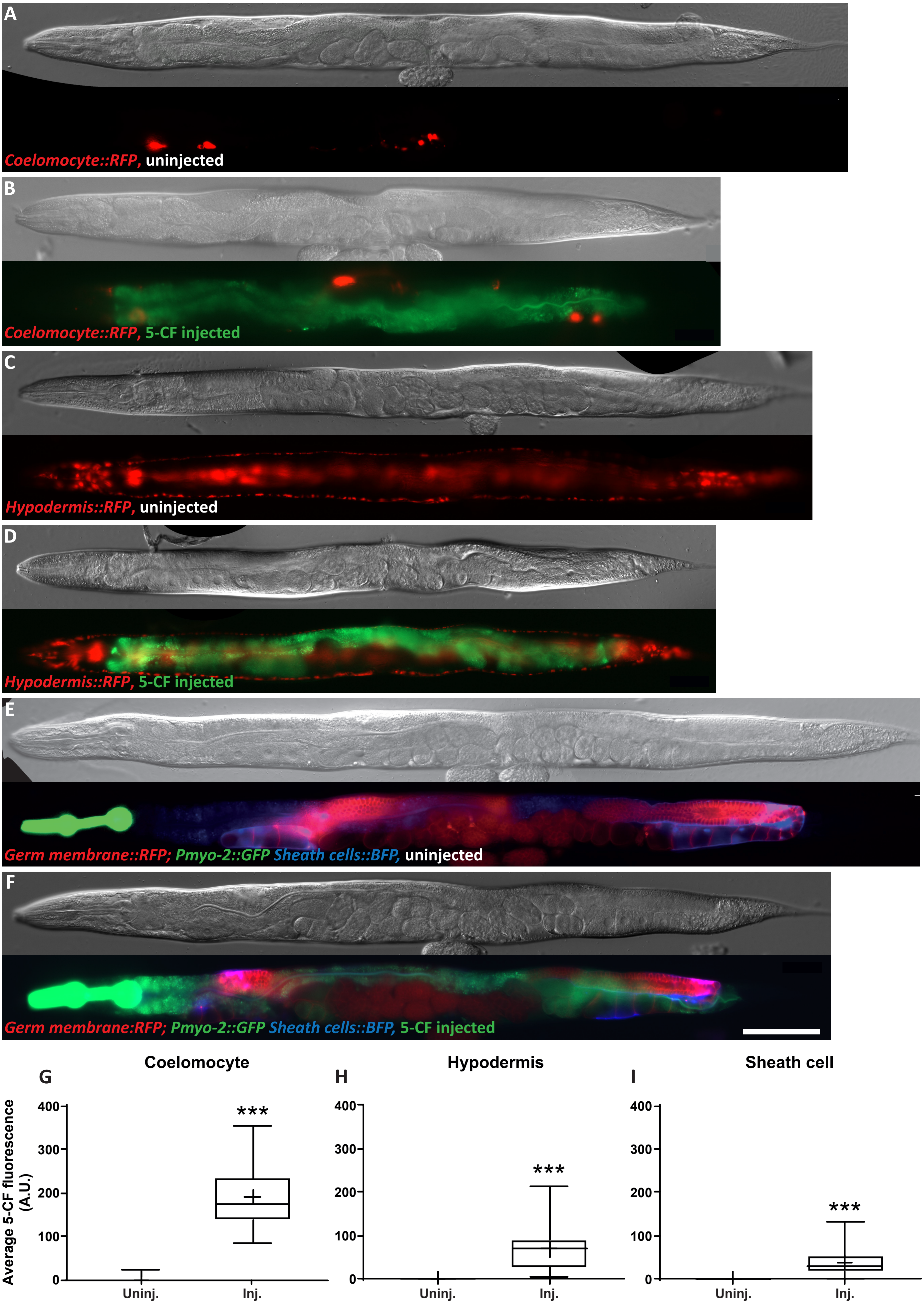

Figure S1. Images and quantification of 5-CF transfer to coelomocytes, lateral hypodermis and proximal sheath cells. (**A-F**) Representative DIC and fluorescence images of adult hermaphrodites expressing tissue-specific fluorescent markers that were or were not microinjected with 5-CF in the intestine, near the vulva. Alleles, *narEx113[coel::RFP; unc-119(+)]*, *foxSi41[Pdpy-7::tomm-20::mKate2::HA::tbb-2 3' UTR]*, *cpSi20[Pmex-5::TagRFP-T::PH::tbb-2 3'UTR + unc-119(+)]*, *narEx110[Plim-7::TagBFP2; Pmyo-2::GFP]*. Scale bar, 100µm. (**G-I**) Average 5-CF fluorescence intensities measured in the labelled indicated tissues (see Fig. 1 and Experimental procedures for details). Anterior and posterior values were averaged together, where applicable. Sample sizes, G: 16, 27; H: 16, 34; I: 12, 11. Statistical tests, G-I: Mann-Whitney.

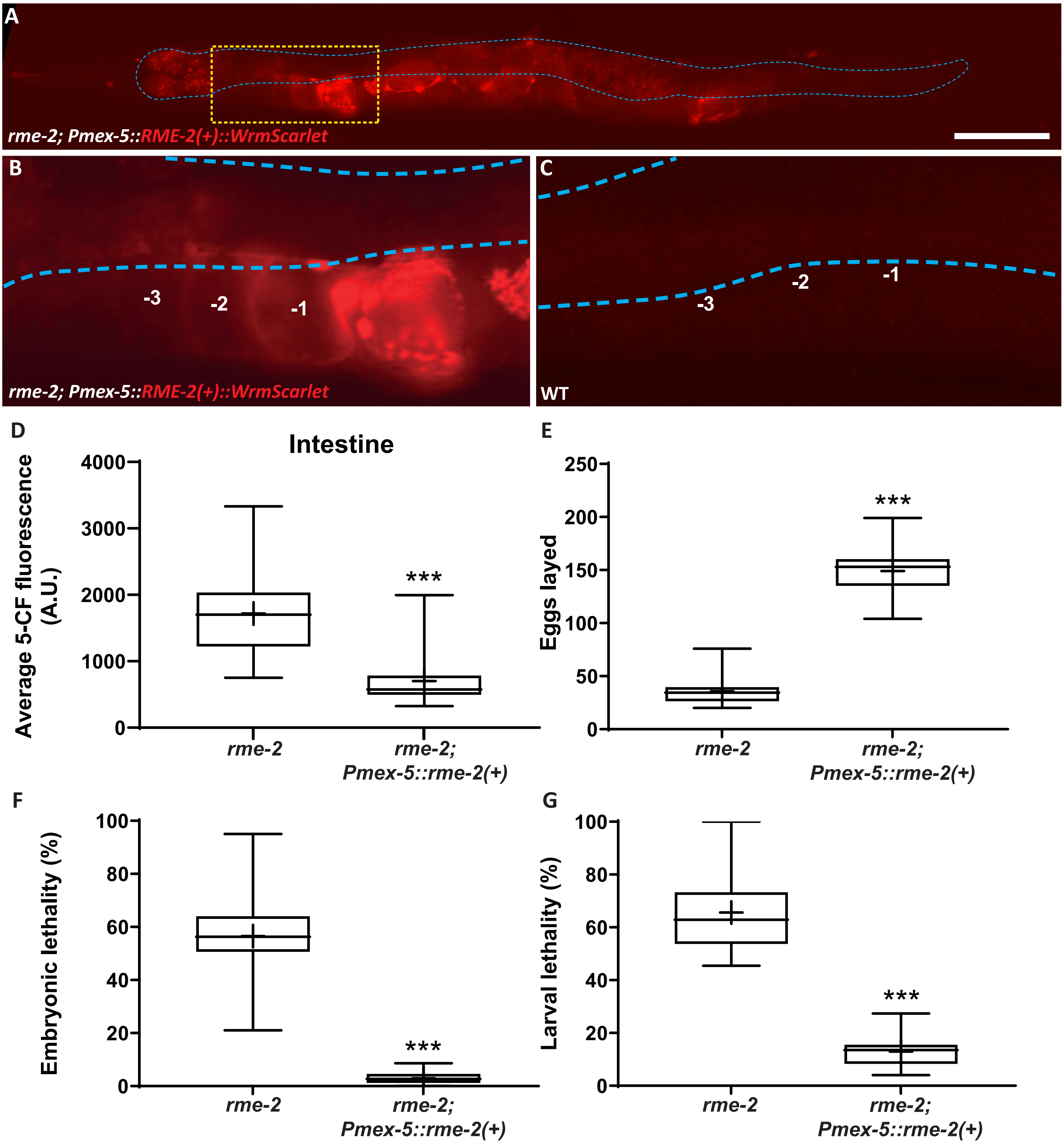

Figure S2. Single-copy rescue of *rme-2(b1008)*. **(A-C)** Representative fluorescence images from (**A-B**) *narSi24[Pmex-5::RME-2::WrmScarlet::rme-2 3'UTR]; rme-2(b1008)* or (**C**) wild-type (non-fluorescent) control adult hermaphrodites. Close-ups of anterior proximal gonads are shown in B-C, acquired using the same parameters. Unexpectedly, in addition to clearly localizing to the membranes of proximal oocytes, some RME-2::WrmScarlet was present in the intestine (delineated by a blue dotted line). As we discuss, we believe this may be the result of GATA motifs within *rme-2* introns as we used its genomic sequence for rescue (see Experimental procedures). Scale bar, 100µm. (**D**) Average intestinal 5-CF fluorescence intensities measured near the injection site in the indicated genotypes. (**E-G**) Average (**E**) number of eggs laid, (**F**) embryonic and (**G**) larval lethality in animals of the indicated genotypes. Sample sizes, D: 30, 31; E-G: 18, 14. Statistical tests, D-E: Mann-Whitney; F-G: Welch's.

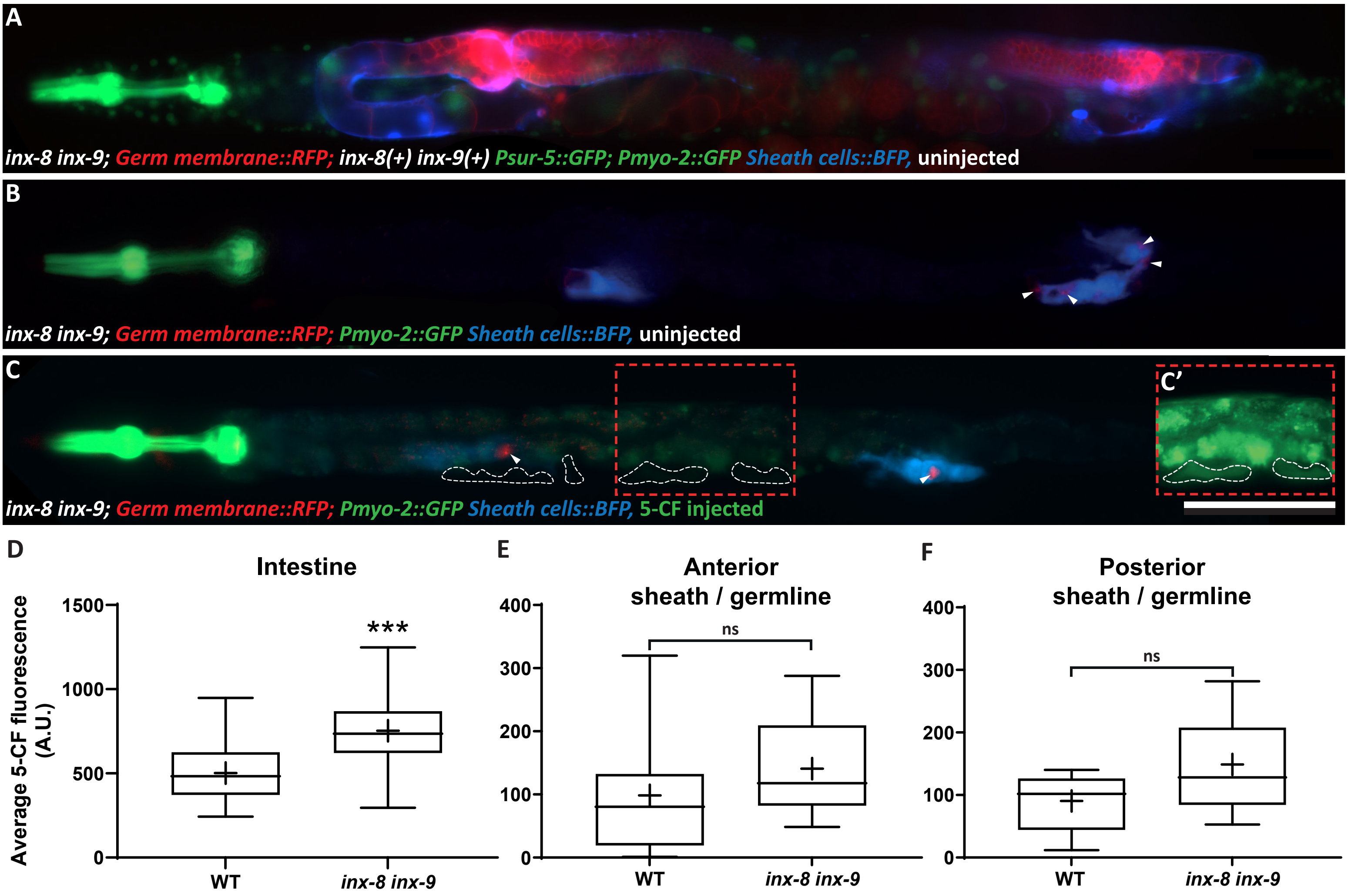

Figure S3. Quantification of 5-CF transfer in *inx-8 inx-9* double mutants. (**A-C**) Representative fluorescence images of animals of the indicated genotypes that were or were not microinjected with 5-CF in the intestine, near the vulva. Where applicable, 5-CF that localized to the pseudocoelom was highlighted by white dashed lines. A region comprising pseudocoelom was enlarged and enhanced in C' to allow visualization of the dimmer signal present in this area. Posterior germline pachytene regions were, where applicable. Scale bar, 100µm. (**D-F**) Average 5-CF fluorescence intensities measured in the indicated tissue(s) of animals of the indicated genotypes. The *inx-8 inx-9* mutants had very few germ cells (labelled red and marked by arrowheads in B-C) that were often comprised within their crumpled sheath cells (labelled blue). We did not observe any obvious differences in the sheath *vs* germ (when present) origin of the faint 5-CF fluorescence signal coming from these tissues and as such, considered them together. We used the germline pachytene values as references for E-F. Alleles, *inx-8(tn1474)*, *inx-9(ok1502)*, *tnEx195[inx-8(+); inx-9(+); Psur-5::GFP]*, *cpSi20[Pmex-5::TagRFP-T::PH::tbb-2 3'UTR + unc-119(+)]*, *narEx110[Plim-7::TagBFP2; Pmyo-2::GFP]*. Sample sizes, D: 23, 24; E: 12, 20; F: 7, 18. Statistical tests, D, F: t-test; E: Mann-Whitney.
